# The right to science in sexual and reproductive health and the legal status of the human embryo

**DOI:** 10.1080/26410397.2025.2524970

**Published:** 2025-06-30

**Authors:** Silke Dyer, Alison B. Edelman, Asha S. George, Tari Turner, Joanna N. Erdman

**Affiliations:** a Professor, Department of Obstetrics & Gynaecology, Groote Schuur Hospital and Faculty of Health Sciences, University of Cape Town, Cape Town, South Africa.; b Professor, Department of Obstetrics & Gynecology, Oregon Health & Science University, Portland, OR, USA; c Professor, School of Public Health, University of Western Cape, Bellville, South Africa; d Professor, School of Public Health and Preventive Medicine, Monash University, Melbourne, Australia; e Professor and MacBain Chair of Health Law and Policy, Schulich School of Law, Dalhousie University, Halifax, NS, Canada

**Keywords:** sexual and reproductive health and rights, assisted reproductive technology, right to science, human embryonic development, policy

## Abstract

Through the examination of scientific evidence related to human embryo development, the aim of this commentary is to support the right to science in sexual and reproductive health, to outline why science supports a legal approach to embryos as the biological material of human life but not as human persons, and to recognise that international human rights law adopts this approach in the protection of sexual and reproductive health and rights.

## Résumé

Grâce l’examen des données scientifiques relatives au développement de l’embryon humain, l’objectif de ce commentaire est de soutenir le droit à la science en matière de santé sexuelle et reproductive, d’expliquer pourquoi la science soutient une approche juridique de l’embryon en tant que matériel biologique de la vie humaine, mais non en tant que personne humaine, et de reconnaître que le droit international des droits humains adopte cette approche dans la protection de la santé et des droits sexuels et reproductifs.

## Resumen

Mediante un estudio de la evidencia científica relacionada con el desarrollo del embrión humano, el objetivo de este comentario es apoyar el derecho a la ciencia en salud sexual y reproductiva, explicar por qué la ciencia respalda un enfoque jurídico con relación al embrión como material biológico de la vida humana pero no como persona humana, y reconocer que el Derecho internacional relativo a los derechos humanos adopta este enfoque en la protección de la salud y los derechos sexuales y reproductivos.

## Introduction

Decades ago, the technology of in-vitro fertilisation supported the development of a human embryo outside the human body. This innovation and many since have led to questions about the legal status of the human embryo, including when human life begins and whether to treat embryos as persons. Science alone cannot answer these questions, but neither can these questions be answered without science.

The global scientific community has played a critical role in informing public debates and shaping public policy on the legal status of the human embryo. We therefore share deep concern about legislative acts and court rulings that misrepresent or ignore scientific evidence when declaring that human embryos are persons.^1–4^ While these events may be localised, they are a symptom of growing, widespread pushback on sexual and reproductive rights.^5^ We are concerned about increasing anti-science governance in sexual and reproductive health, including policymaking that sets aside scientific evidence, and restrictive laws that are based on little or no science. We consider these developments to have common drivers and be of global concern rather than being limited or contained by national borders.

As experts working on the clinical, legal, evidence synthesis and health systems dimensions of sexual and reproductive health and rights, we write (i) to affirm the right to science in sexual and reproductive health, (ii) to share scientific evidence on human embryo development, (iii) to outline why scientific evidence supports a legal approach to embryos as the biological material of human life but not as human persons, and (iv) to recognise that international human rights law adopts this approach in the protection of sexual and reproductive health and rights.

## The right to science in sexual and reproductive health

The realisation of sexual and reproductive health and rights depends on the right to science. Article 15 of the *International Covenant on Economic, Social and Cultural Rights* requires governing authorities to “recognize the right of everyone … to enjoy the benefits of scientific progress and its applications” and “respect the freedom indispensable for scientific research”.^6^ Comprehensively defined by the *Guttmacher-Lancet Commission on Sexual and Reproductive Health and Rights*, and based on the text of historic U.N. agreements, “[s]exual and reproductive health is a state of physical, emotional, mental, and social wellbeing in relation to all aspects of sexuality and reproduction … [including that] all individuals have a right to make decisions governing their bodies and to access services that support that right”.^7^

Additional guidance on the various dimensions of the right to science has been provided in reports by the Special Rapporteurs on cultural rights.^8–10^ The most recent report emphasises that science is a common good, highlights the importance of enhanced dialogue between all stakeholders including at the science–policy interface; and states in Section C.15 that

*“Science liberates people, minds and communities and offers solutions to major challenges that the humanity faces. It enables people to understand the world, dogma to be pushed aside for progress, authority to be questioned, people to communicate, communities to prosper, individuals to attain knowledge and cultures to evolve.”*

Moreover, the right to science is essential to the human right to access quality sexual and reproductive health goods and services, both in the creation of new technologies, and in their effective use and equitable distribution, including emergency contraception, abortion equipment and medicines, and assisted reproductive technologies.^9,10^ Achieving this requires respect for the development and diffusion of science, which entails both a freedom to seek, receive, and impart scientific information, and obliges State parties to refrain from disparagement or deliberate misinformation intended to erode citizen understanding of and respect for science and scientific research.^8^ Scientific knowledge is essential to being an empowered citizen, and scientific freedom is essential to the right of individuals to make decisions governing their bodies in sexual and reproductive health research and care.

Counter-arguments to the importance of science of course exist. These may include that science can produce contradictory, wrong, or biased findings; findings that become refuted; or findings that are misinterpreted. All of this is true. It is the nature of scientific knowledge to evolve, to build new knowledge on prior discoveries, to refine instruments of observation, and to be falsifiable. This makes science stronger, not weaker. Moreover, scientific guidance is increasingly “living” guidance, based on evidence synthesis that is continually updated as new research emerges.

## The science of embryos in human development

Scientifically, human embryos are biological organisms containing unique genetic material. They are the biological material of human life but not human persons. The latter category imposes a single and definitive state on something that is naturally in flux. In biology, the term embryo encompasses the period from completed fertilisation to 56 days post fertilisation (equivalent to 10 weeks of gestational age). It is a period of great transformation at the end of which this organised cellular structure contains organs in their earliest recognisable form. Thereafter, the fetal stage commences (Day >56 post fertilisation until the end of pregnancy).^11,12^ The biological trajectory of a human embryo is highly diverse and uncertain, in both scientific laboratories and human bodies.

Many fertilised eggs (called “zygotes”) do not develop into pregnancies, which begin with implantation, and most do not result in a live birth.^12–15^ Following fertilisation in the Fallopian tube, and depending on the age of the woman, approximately 30% of zygotes fail to implant in the uterus, 30% of implantations cease development prior to any clinical signs of pregnancy, and 10%–15% of clinically established pregnancies miscarry.^13^ The leading cause of these outcomes is aneuploidy (an abnormal number of chromosomes) in the genome of embryos which are not compatible with sustained development.^13,14^ Scientific data from assisted reproductive technology registries demonstrate that the frequency of aneuploidy in human embryos is around 50%.^15,16^

Other biological trajectories include monozygotic twinning, molar pregnancies, as well as major congenital abnormalities incompatible with human development. Monozygotic twinning occurs in approximately 0.5% of births. It is caused by the spontaneous split of one embryo into identical twins which have the same genome but are ontologically different and, therefore, would be different persons upon birth. Unlike an embryo, a human person cannot split itself into two. Complete moles arise from embryos that only have paternal chromosomes. They are characterised by the absence of a fetus and excessive placental growth that can turn into a cancer (choriocarcinoma). All persons can carry a cancer, but they cannot transform into a tumour. Other major abnormalities not compatible with human life, though rare, include the absence of essential organ development or other inborn errors in an embryo.

This scientific evidence of both in-vivo and in-vitro fertilisations, which is based on an extensive body of research, establishes that only a small proportion of embryos develop into a pregnancy and an even smaller proportion into a live birth. Understanding the complexity and many different outcomes of this development makes it easier to recognise why any protection provided to an embryo must be different to that provided to a person. Moreover, following implantation any such protection of the embryo is expressed through the rights and the protection owed to the pregnant woman or person. This understanding supports life-affirming sexual and reproductive health research and care, with immeasurable benefit for human health and well-being. For example, assisted reproductive technology supports people to have children and build families.^17^ Preimplantation genetic diagnosis reduces the burden of inherited diseases. Safe abortion care is essential for saving the lives of millions of women and supports the health and social well-being of millions more.^18^

## Legal approaches to the human embryo

Science cannot decide the legal status of the human embryo, given the relevance of ethical, cultural and religious views on the matter too. However, categorical legal declarations that the human embryo is a person under law – and therefore entitled to the rights and protections of persons within a legal system – ignore current scientific evidence and have led to the withdrawal of sexual and reproductive health care or its provision in ways harmful to people’s health and well-being. This includes restricting women and pregnant persons from making decisions about their bodies, for example, related to access to and use of assisted reproductive technology or by denying the right to quality abortion care. When governing authorities ignore or disregard scientific evidence, it further creates risk and uncertainty for researchers and care providers,^2^ leading to a general divestment from sexual and reproductive health research and practice, a global harm of potentially generational scale.

In contrast, a legal approach that recognises the diverse value of the human embryo as the biological material of human life, scientifically and socially, reflects human reproduction as a complex and uncertain process of development and the constant evolution in our understanding of it. Such an approach can take different forms depending on context, including ethical, cultural, and religious views, and confer different degrees and kinds of protection, but always with respect for the science of human reproduction and the recognition of sexual and reproductive health and research as fundamental human rights. For these reasons, international human rights law adopts this approach in the protection of these rights.

## The status of the embryo in international human rights law

International human rights standards have consistently affirmed that restrictions to sexual and reproductive health care based on the legal status of the embryo-as-person are inconsistent with international law. This affirmation is crucial in a legal system where only human persons are the subjects of fundamental rights to life and health.^19^

In 2012, the Inter-American Court of Human Rights relied on extensive scientific evidence on embryonic development to declare that embryos are not human persons and therefore do not enjoy absolute protection under the right to life.^20,21^ The case involved a ban on in-vitro fertilisation, resulting from a constitutional court ruling based on the right to life of embryos. In its decision, the Inter-American court affirmed that any legal regulation of human embryos must always protect sexual and reproductive rights and provide for the health care means to exercise them.

In 2019, the UN Committee on Economic, Social and Cultural Rights, recognised that any mandated transfer of embryos into the womb of a woman or person after assisted reproductive technology constitutes coercive care in violation of rights to health and equality, which protects bodily integrity in reproductive care, and prevents access to care.^22^ Moreover, in the case brought before the Committee, the embryo transfer was not mandated by law but enforced by clinic staff based on their interpretation of the law, which had formerly mandated the simultaneous transfer of all embryos, regardless of viability or genetic diagnosis and had prohibited embryo freezing. In its decision, the Committee recognised the distinct harms of legal regulation of the human embryo that led to uncertainty and harmful clinical practices and restricted access to care, in violation of the couple’s right to health. The Committee explicitly noted that “the views of society have evolved significantly, and that science and techniques are in a constant state of development” in the field of assisted reproduction, and thus stressed that “[s]tates should update their regulations regularly to harmonize them with their human rights obligations and with the evolution of society and scientific progress” (para 11.4).

While these prominent cases concerned assisted reproductive technology, the human rights standards they articulate also support abortion rights in international law, which align human rights protection with scientific knowledge by requiring the decriminalisation of abortion and access to quality abortion care.^18^

## Conclusion

As affirmed in international human rights law, the science behind human embryo development does not support a categorical legal approach to the human embryo as a human person. Only a legal approach that recognises the complexities of the evolving science in human reproduction, and the advances of this knowledge for the life, health and well-being of all, can protect and promote fundamental human rights. As a core obligation, the right to science thus requires governing authorities to “[p]romote accurate scientific information and refrain from disinformation, disparagement and deliberately misinforming the public in an effort to erode citizen understanding and respect for science and scientific research” (para 52).^9^ This obligation is essential to the full enjoyment of the right to sexual and reproductive health. To achieve this, we call on policymakers and legislators to ensure that policies in sexual and reproductive health are informed by active, systematic, and transparent consideration of relevant, reliable scientific evidence. We ask citizens to commit to holding their representatives to account for this – at the ballot box and through other democratic processes. We strongly encourage researchers to actively engage in decision- and policy-making processes though we recognise that this is not a space in which researchers always feel comfortable. Evidence-based progress will, however, not be achieved unless researchers actively engage in conversations with decision-makers and vice versa. Finally, we appeal to citizens to engage with science and to do so through reliable avenues; and we appeal to researchers to engage with citizens in the prioritisation, conduct and dissemination of research. This mutual engagement will help to both optimise the relevance and impact of sexual and reproductive health research and counter the rising tide of mis- and dis-information. Achieving sexual and reproductive health and rights and the right to science for all will require concerted, proactive effort from all of us.
